# Diagnostic and Prognostic Implications of Caspase-1 and PD-L1 Co-Expression Patterns in Myelodysplastic Syndromes

**DOI:** 10.3390/cancers13225712

**Published:** 2021-11-15

**Authors:** Johannes R. Graf, Stefan Forster, Frido K. Bruehl, Yara Banz, Mahmoud Hallal, Justine Brodard, Vera Ulrike Bacher, Ramanjaneyulu Allam, Christian M. Schürch, Nicolas Bonadies

**Affiliations:** 1 Department of Hematology and Central Hematology Laboratory, Inselspital, Bern University Hospital, University of Bern, 3010 Bern, Switzerland; johannes.graf@lindenhofgruppe.ch (J.R.G.); justine.brodard@insel.ch (J.B.); veraulrike.bacher@insel.ch (V.U.B.); allam.ramanjaneyulu@dbmr.unibe.ch (R.A.); 2 Department for BioMedical Research, University of Bern, 3010 Bern, Switzerland; stefan.forster@dbmr.unibe.ch (S.F.); mahmoud.hallal@live.com (M.H.); 3 Institute of Pathology, Inselspital, Bern University Hospital, University of Bern, 3010 Bern, Switzerland; BRUEHLF@ccf.org (F.K.B.); yara.banz@pathology.unibe.ch (Y.B.); christian.schuerch@med.uni-tuebingen.de (C.M.S.); 4 Department of Pathology and Neuropathology, University Hospital and Comprehensive Cancer Center Tübingen, 72076 Tübingen, Germany

**Keywords:** myelodysplastic syndromes, inflammasome, immune check-point, immune-related biomarkers, diagnosis, prognosis, personalized treatment

## Abstract

**Simple Summary:**

Myelodysplastic syndromes (MDS) originate from mutated hematopoietic stem and progenitor cells. Despite recent advances in genetics, the mechanisms involved in clonal progression remain largely unknown. We performed an exploratory, case-control study to identify immune-related biomarkers with diagnostic and prognostic utility. Our study suggests a combined Casp1/PD-L1 assessment to distinguish reactive conditions from lower- and higher-risk MDS. These immune-related biomarkers may help to personalize immuno-therapies but require further validation in prospective studies.

**Abstract:**

Background: The inflammasome plays an essential role in lower risk MDS and immune subversion, with the up-regulation of immune checkpoint molecules in the progression to higher-risk disease. In this study, we explored the utility of immune-related biomarkers for the diagnosis and prognosis of MDS. Methods: We performed an exploratory, case-control study with 20 randomly selected MDS patients and nine controls with non-inflammatory (*n* = 3) and inflammatory conditions (*n* = 6). Patients were stratified in groups of lower (*n* = 10) and higher risk (*n* = 10) using IPSS-R. For the exploration of inflammasome and immune checkpoint activities, the expression of caspase-1 (Casp1), programmed cell death protein 1 (PD-1) and its ligand (PD-L1) were assessed in bone marrow samples using immunohistochemistry. Results: In multivariate analysis, we observed significant differences for Casp1 but not PD1/PD-L1 expression in our four conditions (*p* = 0.003). We found a discordant co-expression of Casp1/PD-L1 in MDS (rho = −0.41, *p* = 0.07) compared with a concordant co-expression in controls (rho = 0.64, *p* = 0.06). Neutrophil counts correlated directly with Casp1 (rho = 0.57, *p =* 0.009) but inversely with PD-L1 expression (rho = −0.58, *p* = 0.007). Conclusion: We identified characteristic discordant co-expression patterns in lower- (Casp1_high_/PD-L1_low_) and higher-risk MDS (Casp1_low_/PD-L1_high_), contrasting with concordant patterns in the non-inflammatory (Casp1_low_/PD-L1_low_) and inflammatory conditions (Casp1_high_/PD-L1_high_). Further validation is warranted in larger, prospective studies.

## 1. Introduction

Myelodysplastic syndromes (MDS) are heterogeneous diseases originating from somatically mutated, hematopoietic stem and progenitor cells (HSPCs). Their clinical features comprise inflammation, dysplasia, cytopenia and a propensity to evolve towards acute myeloid leukemia (AML). Ineffective hematopoiesis is the hallmark of MDS, which is characterized by increased proliferation of dysplastic HSPCs, followed by premature cell death in the bone marrow (BM) and cytopenia in the peripheral blood (PB) [1,2]. Despite recent advances in the genetic characterization of MDS, the mechanisms involved in clonal progression from lower to higher risk disease remain largely unknown. The disease-based risk of MDS patients is currently assessed by the international prognostic scoring system (IPSS-R), which is based on the degree of cytopenia, percentage of blasts in PB and BM, and cytogenetic alterations [3,4]. In recent years, molecular diagnostics has shown its utility for the diagnosis, prognosis and prediction of treatment response [5]. In contrast, immune-related biomarkers remain poorly characterized but are of increasing interest, due to their potential implications as targets in MDS treatment.

On the molecular level, somatic mutations in HSPCs induce activation of the inflammasome through the NACHT, LRR and PYD domains-containing protein 3 (NLRP3). This leads to caspase-1 (Casp1)-induced pyroptotic cell death accompanied by the activation of beta-catenin, which is involved in the clonal dominance of mutated and the exhaustion of normal HSPCs [1,2]. This biological phenomenon is supposed to be the basis for a vicious circle fueling clonal evolution and provides potential novel treatment targets [6]. So far, allogenic hematopoietic stem cell transplantation (allo-HSCT) remains the only curative treatment option for eligible MDS patients. However, the majority of higher risk MDS patients are elderly and not fit enough for this intensive treatment. Most patients receive palliative therapy with hypomethylating agents (HMA) and all will eventually progress to AML [7]. Therefore, there is an unmet need in identifying new treatment options and the modulation of immune-related effector mechanisms seems to be a promising field of further clinical investigation [7,8,9,10,11,12]. 

Our study group and others have previously shown that 10–30% of MDS patients suffer from a broad variety of autoimmune and autoinflammatory manifestations [13,14,15]. However, it remains currently unclear whether these immunological phenomena are irrelevant by-stander effects or directly involved in shaping clonal evolution. Recent studies have highlighted the important role of the adaptive and innate immune system in the disease initiation and progression of MDS [6]. An increase of CD8+ and CD4+ T-cells as well as NK cells combined with a decrease of regulatory T cells (Treg) has been observed in the BM of lower-risk MDS patients, while CD8+ and CD4+ T cells and NK cells are decreased and Tregs are increased in higher risk disease [16]. In addition, the negative co-stimulatory T-cell receptor programmed cell death-1 (PD-1) and its ligands, PD-L1 and PD-L2, were found to be overexpressed in the progression from lower to higher risk MDS [17,18]. The interactions of PD1 with PD-L1 or PD-L2 are immune checkpoints (IC) that suppress cellular immune effector mechanisms and are frequently associated with progressive disease in a variety of cancers [19]. The transition from a tumor engaging to a permissive immunological state is usually referred to as immune subversion [20]. In lymphoma, increased PD1/PD-L1 expression has been described as a consequence of inflammasome activation [21,22,23]. However, the interaction of inflammasome activation with IC molecules in MDS as well as their potential implications for diagnosis, prognosis and treatment remain currently unclear. To this end, we performed an exploratory study to assess the utility of a combined analysis of Casp1 and PD-1/PD-L1 expression as potential immune-related biomarkers in diagnosis and prognosis of MDS patients.

## 2. Methods

### 2.1. Study Design 

We performed an investigator-initiated, single-center, retrospective, case-control study. This study was based on the Swiss MDS Registry and Biobank platform, which was approved by the competent local ethics committee (2016-01917, 2017-02299, 2017-00699). All MDS patients provided their informed consent to the study.

### 2.2. Selection of Patient and Control Samples

In our cohort, we randomly selected 20 MDS patients and nine controls, with bone marrow (BM) trephine biopsy samples collected between 05/2008 and 03/2016, as well as at an available clinical follow-up until 01/2018. All relevant clinical data of MDS patients was extracted from the Swiss MDS Registry database and missing data was completed using the local clinical information system. In 15 MDS patients, the BM biopsies used for immunohistochemistry (IHC) analysis were collected at diagnosis and in five patients at follow-up. Controls underwent BM assessment for a variety of indications and had no evidence of clonal hematological disorders. All BM biopsies of patients and controls were stored and processed at the Institute of Pathology, University of Bern. For the external validation of Casp1, PD1 and PD-L1 expression in MDS patients, we searched in the following publicly available RNA- and protein-expression databases: Gene Expression Omnibus [24]; Human Protein Atlas [25]; EMBL-EBI Expression Atlas [26].

### 2.3. Definition of Disease and Patient Characteristics

MDS patients were categorized according to the 2016 revision of the WHO classification [27,28] and risk stratified in lower risk (*n* = 10) and higher risk (*n* = 10) using an IPSS-R score > 4 as cut-off [4,29]. Patient-based risk was assessed by the MDS-Comorbidity Index (MDS-CI) [30] and treatment histories with relevant clinical endpoints (see below) were collected. Controls had no morphological signs of BM involvement and were divided into two groups; non-inflammatory conditions without (*n* = 3) and inflammatory conditions with associated systemic inflammation (*n* = 6). Assessment of relevant systemic inflammation was based on clinical information and laboratory values (Table 1).

### 2.4. Definition of Clinical Endpoints 

Time to treatment (TTT), progression-free survival (PFS), leukemia-free survival (LFS), and overall survival (OS) were used as clinical endpoints. TTT was defined as time from diagnosis until first administration of a disease-modifying treatment (DMT: azacitidine, lenalidomide, cytarabine/idarubicin, cyclosporine A, thymoglobulin, hydroxyurea), including allo-HSCT. PFS was defined as time from diagnosis until progression from lower to the higher risk MDS, progression to AML or death from any cause. LFS was defined as time from diagnosis until progression to AML or death from any cause. OS was defined as time from diagnosis until death from any cause. 

### 2.5. Reagents 

Casp1 expression was used as surrogate for inflammasome and PD-L1/PD-1 expression for IC activity. Expression of Casp1 was assessed by IHC using Caspase-1 p20 C-15 antibody from Santa Cruz Biotechnology (sc-1780) on a BOND III staining platform (Leica Biosystems AG). Slides were pretreated with tris-based buffer (20 min at 95 °C), followed by the primary antibody (30 min at RT, diluted 1:500). For the check-point inhibitor PD-L1 antibody (790-4905) from Roche Diagnostics, we used a Benchmark Ultra staining platform from Ventana Roche. Slides were pretreated with tris-based buffer (Ultra CC1, 64 min at 100 °C), followed by incubation with the primary antibody (16 min at 37 °C), OptiView Linker (8 min) and OptiView HRP Multimer (8 min). For the PD-1 antibody (315M-94) from Marque, we used a BOND III staining platform (Leica Biosystems AG). Slides were pretreated with EDTA based buffer (pH 9.0) and epitope retrieval solution, followed by the primary antibody (15 min at RT, diluted 1:150), post-primary AB (8 min) and polymer (8 min). All slides were counterstained with hematoxylin.

### 2.6. Data Collection, Scoring and Analysis Plan

Immunohistochemistry staining evaluation was performed by two pathologists in-training (SF, FB), blinded for the clinical information, and supervised by two board-certified pathologists specialized in hematopathology (YB, CMS). Calculation was made on the basis of the Histo-score (H-score), a combined quantitative and qualitative score based on number of positive cells and the intensity of staining (graded as: 0 = non-staining; 1 = weak; 2 = median; or 3 = strong) [31,32,33]. Casp1 and PD-L1 were scored independently by both raters in two independent rounds and average H-scores were calculated. Differences in H-score for Casp1, PD-L1 and PD-1 between lower and higher risk MDS were investigated. Patients were stratified in high or low expression using the corresponding median H-score as cut-off. H-scores were used for correlations with relevant laboratory values, including neutrophils, monocytes, hemoglobin, platelets, BM cellularity, BM blasts, and C-reactive protein (CRP), as well as for survival endpoints (TTT, PFS, LFS, OS).

### 2.7. Statistical Analysis

Statistical analysis was performed using R studio’s basic packages [34,35]. Patient and disease characteristics were compared between the clinical groups using the Mann–Whitney U test for ordinal scaled data and the Fisher’s exact test for contingency tables. For univariate analysis, H-scores were compared between the two clinical risk groups using the Mann–Whitney U test. For multivariate analysis, H-scores were compared using the Kruskal–Wallis test for ordinal scaled data with a subsequent Dunn test with Bonferroni correction. Spearman’s rank was used for correlation of H-scores with clinical and laboratory characteristics. Time-dependent variables were compared using Kaplan–Meier estimator with log-rank test for survival rates, censored for allo-HSCT at time of transplantation. Unless stated otherwise, *p* values < 0.05 were considered significant. The pROC R package was used for the calculation of specificity, sensitivity positive predictive value, and negative predictive value, as well as the receiver operating characteristic (ROC) and the determination of the area under the curve (AUC) with a 95%-confidence interval [36]. The ggplot2 R Package was used to create the Swimmer’s plot [37].

## 3. Results

### 3.1. Baseline Characteristics of MDS Patients and Controls

We included 20 MDS patients and nine controls in our study. Lower and higher risk MDS patients were equally distributed and controls comprised three individuals with non-inflammatory and six with inflammatory BM conditions (overview in Table 1, details in Table S1). Before inclusion, eleven MDS patients underwent regular transfusions; ten received red blood cells (RBC) and one platelet concentrates (PC). One higher risk MDS patient received two cycles of lenalidomide four months before inclusion. MDS patient characteristics were similar in both risk groups and numbers generally too low to identify small differences between higher and lower risk. The exceptions were a tendency to higher neutrophil (mean ± SD: 2.32 ± 1.53 G/L vs. 1.36 ± 1.53 G/L, *p* = 0.14) and significant lower BM blasts counts (median with range: 3% (0–4%) vs. 9% (2–15%), *p* = 0.03) of lower compared with higher risk, respectively. 

The three non-inflammatory BM conditions comprised samples from individuals with recovery after a transfusion reaction, localized (extramedullary) follicular lymphoma and solitary (extramedullary) plasmocytoma. The six inflammatory conditions included samples from patients with a variety of severe systemic inflammatory disorders without morphological alterations of the BM (Hodgkin lymphoma, pulmonary adenocarcinoma, diffuse large B-cell lymphoma, post-transplant lymphoproliferative disorder, cerebral toxoplasmosis secondary to acquired immune deficiency syndrome (AIDS), and multicentric Castleman disease). Neutrophil counts (8.55 ± 7.75 10^9^/L vs. 4.61 ± 4.61 10^9^/L), CRP (68.2 ± 47.1 mg/L vs. 3.0 ± 3.0 mg/L), and ferritin levels (895.7 ± 568.9 µg/L vs. 484.0 ± 484.0 µg/L) tended to be higher in the inflammatory compared with non-inflammatory conditions, but the numbers were beneath significance. The BM showed normal cellularity and no excess of blasts in both control conditions.

### 3.2. Co-Expression Pattern of Casp1/PD-L1 in MDS Patients and Controls 

**Figure 1:**
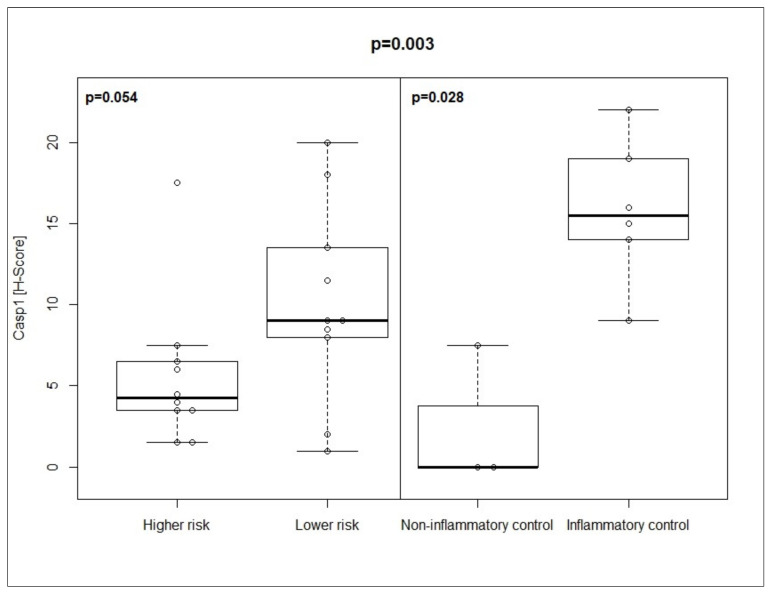
Expression of Casp1 in MDS patients and controls. H-scores are shown as median with ranges. *p*-values were calculated with Mann–Whitney U test for univariate and Kruskal–Wallis test for multivariate analysis. Significance was determined at *p <* 0.05. Cohen’s effect size r was large at 0.83.

**Figure 2:**
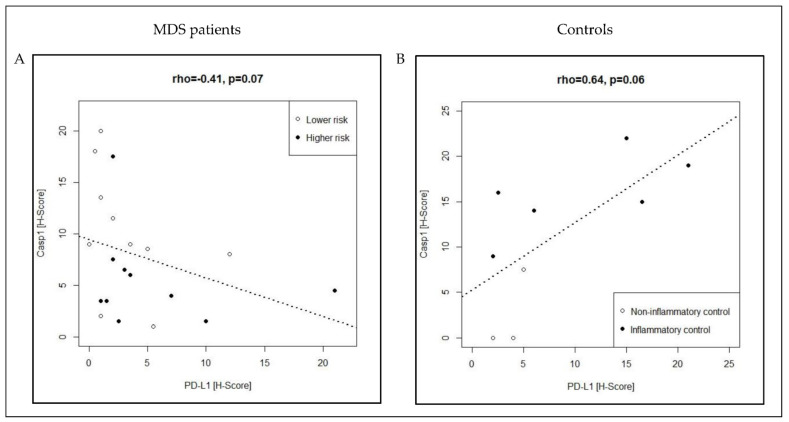
Relevant correlations between immune-related biomarkers in MDS patients (**A**) and controls (**B**). Regression analysis was performed with Spearman’s rank correlation and significance determined using Bonferroni correction. Refer also to Table S2.

In univariate analysis, Casp1 expression tended to be higher in lower- compared with higher-risk MDS (median H-score: 9 vs. 4.25; *p* = 0.054). Moreover, expression was significantly higher in inflammatory compared with non-inflammatory conditions (median H-score 15.5 vs. 0; *p* = 0.028) (Figure 1; Table 2). Multivariate analysis identified significant differences for Casp1 expression in the four conditions (*p* = 0.003), notably between higher risk MDS (Casp1_low_) and other inflammatory conditions (Casp1_high_) and between non-inflammatory (Casp1_low_) and inflammatory conditions (Casp1_high_) (*p adj = 0.01 for each*) (Table 2). Neither in uni- nor in multivariate analysis could significant differences be determined for PD1 or PD-L1 expression. Representative histomorphological pictures for Casp1 and PD-L1 expression in the four groups can be found in Figures S1 and S2. Using regression analysis, we identified a negative (inverse) correlation between Casp1 and PD-L1 (rho = −0.41, *p = 0.07*) and a positive correlation between PD-L1 and PD1 (rho = 0.42, *p = 0.07*) in MDS patients (Figure 2A; Table S2). In contrast, we found a positive correlation between Casp1 and PD-L1 (rho = 0.64, *p = 0.06*) in controls (Figure 2B; Table S2).

In conclusion, our observations suggest a discordant co-expression pattern in MDS patients with Casp1_high_/PD-L1_low_ in the lower- compared with Casp1_low_/PD-L1_high_ in higher-risk, respectively. Moreover, we identified a concordant co-expression pattern in the controls with Casp1_low_/PD-L1_low_ in the non-inflammatory and Casp1_high_/PD-L1_high_ in the inflammatory BM conditions, which was distinct from the discordant expression in MDS patients.

### 3.3. Association of Casp1/PD-L1 Expression with Neutrophil and Monocyte Counts in MDS Patients

**Figure 3:**
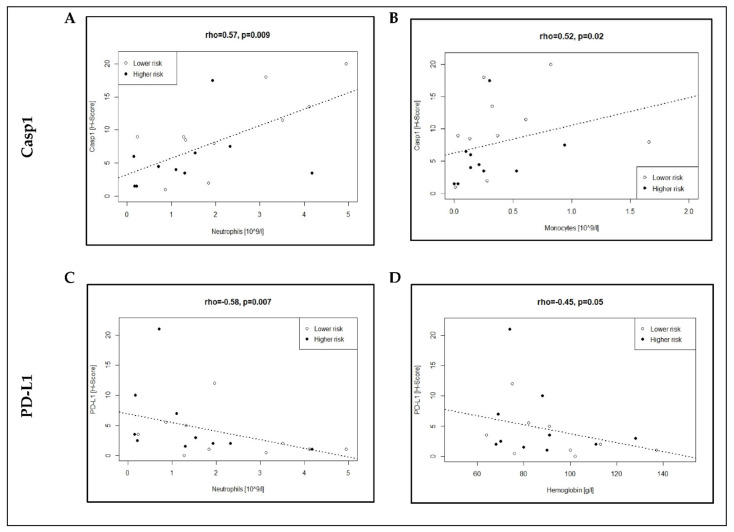
Relevant correlations between immune-related biomarkers Casp1 (**A**,**B**) and PD_L1 (**C**,**D**) and blood counts in MDS patients. Regression analysis was performed with Spearman’s rank correlation and significance determined using Bonferroni correction. Refer also to Table S3.

Using univariate regression analysis, we identified in MDS patients a positive correlation of Casp1 expression with neutrophil (rho = 0.57, *p =* 0.009) as well as monocyte counts (rho = 0.52, *p* = 0.02), in contrast to a negative correlation of PD-L1 expression with neutrophil counts (rho = −0.58, *p* = 0.007) and hemoglobin concentration (rho = −0.48, *p =* 0.045) (Figure 3; Table S3). Only the correlations of neutrophils with Casp1 and PD-L1, respectively, maintained significance after correction for multiple testing. No significant correlation was found in univariate analysis of Casp1, PD-1 and PD-L1 with BM blasts, cellularity, or CRP, suggesting less relevant interactions with these factors in MDS patients (Table S3). 

In summary, these observations in MDS patients further support the finding of a discordant expression of the immune-related biomarkers in lower- (Casp1_high_/PD-L1_low_) and higher-risk (Casp1_low_/PD-L1_high_) disease, as blood counts generally correlate with disease state. 

### 3.4. Association of Immune-Related Biomarkers with Clinical Endpoints in MDS Patients 

We further investigated the association of immune-related biomarkers with relevant clinical endpoints in MDS patients. The median follow-up time was 623 days, with 649 (range 107–1799) and 471 (range 15–3576) days for lower- and higher-risk patients, respectively (Table 3). As expected, we found a significantly longer time to treatment (TTT: 289 vs. 55.5 days; *p =* 0.04), but only trends for prolonged survival endpoints (PFS: 429 vs. 162 days, LFS: 951 vs. 143.5 days and OS: 955 vs. 170.5 days) in lower- compared with higher-risk disease (Table 3; Figure S3). Using the corresponding median H-scores as cut-offs (Casp1: 7, PD1: 3, and PD-L1: 2.25), we could not identify any significant survival differences between low- and high-expressing groups (Table S4). In conclusion, the investigated MDS patient numbers were too low to discriminate any significant impact of our immune-related biomarkers on relevant clinical endpoints.

### 3.5. Casp1/PD-L1 Co-Expression Patterns in Individual Clinical Courses of MDS Patients

**Figure 4:**
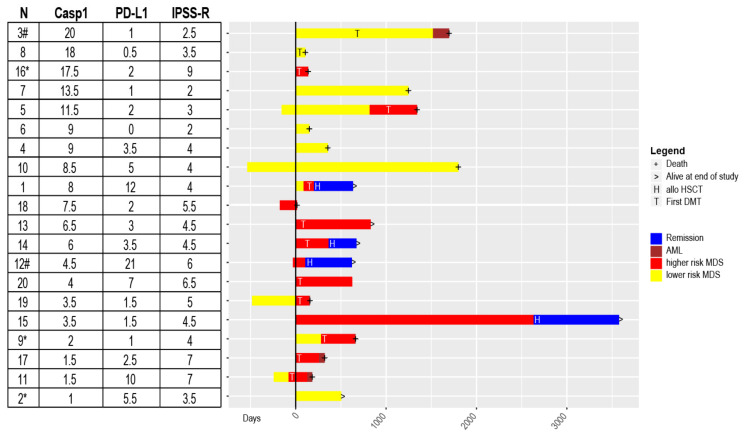
Clinical course of MDS patients ranked according to Casp1 expression. Numbers are according to Supplementary Table S1 with patient numbers [N], Casp1 and PD-L1 [H-scores] expression and International Prognostic Scoring System revised [IPSS-R], shown in the table on the left. Patients were ranked according to the Casp1 expression. The general pattern of Casp1low in higher-risk and Casp1high in lower-risk disease is evident. The five patients that were not included at diagnosis have bars in the negative time-axis. I.e. patient 11 received 2 cycles of lenalidomide four months before inclusion, all others were DMT-naïve. Only patients undergoing allo-HSCT achieved durable remission. IHC pictures of patients 3 and 12 with # are shown in Figures S1 and S2, respectively. Patients 2, 9, 16 with * had unexpected patterns and were investigated in more detail.

Based on our observation that Casp1/PD-L1 co-expression may be associated with disease stage, we ranked our MDS patients according to the Casp1 expression level and visualized the individual clinical courses using swimmer’s plot (Figure 4, detailed clinical information can be found in Table S1). This representation allowed us to highlight the general pattern of Casp1_high_ in lower-risk and Casp1_low_ in higher-risk patients, as stated above. However, we identified three patients, who did not match to the expected pattern and were investigated in more detail:

Patient #2 (28 years, male) with Casp1_low_ (H-score 1) and PD-L1_high_ (H-score 5.5) was diagnosed with lower-risk MDS (IPSS-R 3.5 points). He presented with a bone marrow failure syndrome with hypoplastic MDS caused by a telomeropathy in the context of a germ-line predisposition syndrome for myeloid malignancies. The most likely cause for this unexpected pattern was the hypocellular BM.

Patient #9 (59 years, male) with Casp1_low_ (H-score 2) and PD-L1_low_ (H-score 1) was diagnosed with lower-risk MDS with a borderline IPSS-R score (4 points) and hypocellular BM. He transformed into higher-risk MDS within one year, which was generally more reminiscent of higher-risk disease state. Like patient #2, the hypocellularity may explain this pattern. Only patient #2 and #9 had a hypocellular BM in the trephine biopsies.

Patient #16 (74 years, male) with Casp1_high_ (H-score 17.5) and PD-L1_low_ (H-score 2) was diagnosed with higher risk MDS (IPSS-R 9 points) with low CRP (4 mg/L), normal neutrophils, excess of blasts 2 and complex karyotype. This patient emerged as a clear outlier in the boxplot of Figure 1 and the patient had no signs of relevant inflammation at diagnosis. The only features that distinguished this patient form the others were the normal neutrophil count combined with a complex karyotype at diagnosis. 

### 3.6. Co-Expression Pattern of Casp1/PD-L1 Might Be Useful as Classifier for MDS Disease State

To investigate the potential prognostic utility of the assessment of Casp1 expression, we performed a receiver operating characteristics (ROC) analysis. We found that, using a cut-off H-score of 7.8, Casp1_high_/Casp1_low_ classified MDS cases correctly in lower/higher risk with a specificity of 90%, sensitivity of 80%, positive predictive value of 88% and negative predictive value of 82%, (AUC 76%: 95% CI: 50.8–100%) (Figure S4). As patient numbers were low for definitive conclusions, this warrants further investigations in prospective studies.

## 4. Discussion

Growing interest is emerging for the identification of immune-related biomarkers to individualize assessment of immune-states in MDS patients. This development is mainly based on the recent discoveries of the NLRP3-based inflammasome activation as well as immune checkpoint (IC) molecules that are involved in the establishment and progression of the disease [2,6,38]. Information on immune states is relevant, and the success of therapeutic interventions is dependent on a dynamic and individual immunogenic context [39]. Ongoing clinical trials are investigating the efficacy and safety of different IC inhibitors in combination with HMA in lower- and higher-risk MDS patients [7,40]. Moreover, modulators of the NLRP3-inflammasome activation pathway are investigated in pre-clinical studies and about to enter clinical trials in MDS as novel targeted treatments in lower-risk disease [22,23]. Evidence is emerging from basic research that these pathways for the innate and adaptive immunity are interconnected [1,2,6]. However, to the best of our knowledge, systematic investigations on the co-expression pattern of IC molecules and inflammasome activity, as well as their clinical implications are lacking in MDS patients. 

In this study, we explored the utility of a combined analysis of Casp1, PD-L1 and PD-1 as potential immune-related biomarkers for the diagnosis and prognosis of MDS patients. Whereas the expression pattern of Casp1 in lower- (high expression) and higher-risk (lower expression) patients has been previously described by Basiorka et al. [38], our data suggests the diagnostic and prognostic implications of a combined Casp1 and PD-L1 assessment, which provides additional utility beyond the isolated assessment of inflammasome activity. We observed a discordant co-expression pattern in MDS patients with Casp1_high_/PD-L1_low_ in lower- compared with Casp1_low_/PD-L1_high_ in higher-risk MDS, respectively, with potential prognostic utility. In contrast, the co-expression pattern in the controls was concordant with Casp1_low_/PD-L1_low_ in non-inflammatory and Casp1_high_/PD-L1_high_ in inflammatory BM conditions, which provides additional diagnostic information beyond MDS risk-classification. We found a direct correlation of Casp1 expression with neutrophil and monocyte counts and an inverse correlation of PD-L1 expression with neutrophil counts and hemoglobin concentration. These correlations further support our observed expression pattern in MDS of lower and higher risk, considering that these blood counts usually correlate with disease state. The expressions of our investigated immune-related biomarkers are not only dependent on disease state, but also on individual immune states and may explain some paradoxical observations reported with IC inhibitor treatments [39]. The Casp1 expression pattern we found in our MDS patient cohort is in line with previously published flow cytometry-based results, where higher Casp1 expression was reported in mononuclear cells in lower- compared with higher-risk MDS patients [41]. Other clinical investigations reported consistently overexpression of PD-L1 and PD-L2 at progression from lower- to higher-risk MDS, which aligns well with our observation [17,18]. These findings underline that a dynamic and stage-dependent transition from a tumor engaging to a permissive state (immune subversion) plays a role in MDS patients which forms the basis of ongoing clinical trials with IC inhibitors and modulators of inflammasome activity [11,42]. We performed a search of publically available RNA and protein databases, for the external validation of Casp1, PD1, and PD-L1 expression in MDS patients. The following RNA- and protein-expression databases were screened: Gene Expression Omnibus [24]; Human Protein Atlas [25]; and EMBL-EBI Expression Atlas [26]. We identified two relevant RNA datasets reporting Casp1, PD1, and PD-L1 expression in different MDS subgroups and controls (GEO DataSet Browser on nih.gov, accessed on 19 October 2021: GDS3795, GDS2118) [43,44]. RNA-expression analysis showed heterogeneous results for higher- and lower-risk MDS patients and could not recapitulate the published patterns of Casp1 and PD-L1 protein expression [17,18,38,41]. This is not surprising, as mRNA expression has notoriously poor correlation with protein expression and should not be used for inferences. Studies have shown that RNA and protein expression may correlate only in one third of cases and performed better using differentially expressed RNA [45,46]. An untargeted analysis of protein expression (proteome) would be desirable for the external validation of our pattern; however, to the best of our knowledge, such a publically accessible dataset of cellular proteins is currently not available with which to compare differential expression in lower- and higher-risk MDS patients.

The distinct co-expression pattern observed in inflammatory conditions (concordant) and MDS (discordant) co-expression pattern is interesting and hypothesis-generating. However, the potential mechanisms involved in either immune activation (Casp1_high_/PD-L1_low_) or inhibition (Casp1_low_/PD-L1_high_ in higher-risk MDS) remain to be further explored. Casp1 is established as a driver of pyroptosis in inflammation and oncogenesis [47,48]. Our observations of down-regulation of Casp1 and up-regulation of PD-L1 at the transition from lower to higher risk open some relevant questions regarding potential pitfalls in drug targeting [1,38]. For instance, in malignant lymphoma, NLRP3-inflammasome activation correlated with up-regulation of PD-L1, which contrasts with the discordant pattern in our MDS patients and is more reminiscent of an inflammatory condition. In this lymphoma model, inhibition of inflammasome activity restored immune-effector functions, but the combined inhibition of the inflammasome and PD-1/PD-L1 was deleterious and caused progressive disease [23]. This observation suggests that the timing of the intervention on immune-effectors is context-dependent and inappropriate modulation can be potentially counterproductive. Therefore, the reliable assessment of immune states with immune-related biomarkers (immunometer) represents an unmet need to optimize and individualize immuno-therapies. 

The detailed analysis of individual disease courses using swimmer’s plots underlined the pattern of Casp1_high_ in lower and Casp1_low_ in higher risk disease. However, as with most biomarkers, potential confounding factors need to be considered, such as inflammatory conditions with neutrophilia or monocytosis (biased towards higher expression), hypoplastic BM (biased towards lower expression), and potential other biological features that need to be further identified in larger prospective cohorts. We found a promising sensitivity of 80% and specificity of 90% in classifying lower/higher risk disease using Casp1 expression. However, the patient numbers were too low to assess the full range of variability and to draw definitive conclusions. Further validations within larger and prospective studies are warranted and it would be of particular interest to investigate, if immune-related biomarkers correlate with mutational profiles and improve prognostic risk assessment, especially in lower- or intermediate-risk MDS patients. 

Beside the low number of patients, our study was limited by the retrospective design, the semi-quantitative assessment of biomarker expression and the limited information on the cell-type specific expression using IHC. Moreover, the turn-around time of IHC is inconvenient to rationalize further diagnostic management of MDS patients in a timely fashion. Nonetheless, we believe that the combined assessment of Casp1/PD-L1 is novel and our described pattern is sufficiently supported by previously published data from the single markers Casp1 and PD-L1/PD-L2, respectively, as described above [17,18,38,41]. Moreover, we found inverse correlation in MDS and a direct correlation in controls, which could be of additional clinical relevance. However, further validation is required and we are currently planning a prospective, flow cytometry-based analysis to confirm the diagnostic and prognostic utility of our immune-related biomarkers (including PD-L2, CD47, CD70 and TIM3) in a much larger cohort of patients with unclear cytopenia, MDS and related disorders.

## 5. Conclusions

Our study suggests a diagnostic and prognostic utility of Casp1 and PD-L1 co-expression patterns in distinguishing between inflammatory conditions (Casp1_high_/PD-L1_high_), lower- (Casp1_high_/PD-L1_low_) and higher-risk MDS (Casp1_high_/PD-L1_low_). The novelty in our report lies in the combined assessment of the inflammasome activity (Casp1) and the expression of immune-checkpoints (PD-L1) with potential future clinical implications. Both are relevant immune effectors of innate and adaptive immunity, respectively, and currently investigated as potential drug targets in MDS. Moreover, the combined assessment of these immune-related biomarkers has not yet been established and provides the opportunity to identify those MDS patients that have undergone immune subversion from a tumor-engaging to a tumor-permissive immunological state, which will allow personalizing targeted immuno-therapies. Further validation of our proposed immune-related biomarkers is warranted by flow cytometry or other single-cell based approaches in larger, prospective studies.

## Figures and Tables

**Table 1:** Characteristics of MDS patients and controls.

Characteristics	MDS Patients			Controls		
	Lower Risk (*n* = 10)	Higher Risk (*n* = 10)		Non-Inflammatory Controls (*n* = 3)	Inflammatory Controls (*n* = 6)	
**Gender**						
Male	8 (80)	6 (60)	*ns*	2 (66)	6 (100)	*ns*
Female	2 (20)	4 (40)	*ns*	1 (33)	0 (0)	*ns*
**Age**	71 (28–79)	69.5 (21–76)	*ns*	59 (58–60)	56 (18–67)	*ns*
**Blood counts**						
hemoglobin [g/L]	99.5 (28.8)	86.9 (19.6)	*ns*	131.5 (30.5)	104 (25.6)	*ns*
thrombocytes [10^9^/L]	121 (132.7)	76.4 (73.5)	*ns*	250.5 (84.1)	278 (172.3)	*ns*
neutrophils [10^9^/L]	2.32 (1.53)	1.36 (1.24)	*p =* 0.14	4.61 (4.61)	8.55 (7.75)	*ns*
monocytes [10^9^/L]	0.45 (0.49)	0.26 (0.28)	*ns*	0.37 (0.37)	1.92 (1.92)	*ns*
**Blood chemistry**						
**CRP [mg/L]**	16.2 (19.7)	26.3 (45)	*ns*	3.5 (0.7)	68.2 (47.1)	*ns*
creatinin [μmol/L]	81.6 (14.5)	85 (27.9)	*ns*	73.0 (73.0)	117.0 (77.3)	*ns*
ASAT [U/L]	29.4 (16.9)	20.4 (5.9)	*ns*	44.0 (44.0)	26.3 (7.5)	*ns*
LDH [U/L]	431.6 (136.2)	479.7 (107.3)	*ns*	378.0 (378.0)	486.2 (179.2)	*ns*
ferritin [μg/L]	556.2 (444.6)	395.4 (338.1)	*ns*	484.0 (484.0)	895.7 (568.9)	*ns*
**Bone marrow**						
dysplastic myelopoiesis	9 (90)	10 (100)	*ns*	0 (0)	0 (0)	*ns*
dysplastic megakaryopoiesis	8 (80)	9 (90)	*ns*	0 (0)	0 (9)	*ns*
dysplastic erythropoiesis	9 (90)	8 (80)	*ns*	0 (0)	2 (33)	*ns*
fibrosis, grade 2 or higher	0 (0)	1 (10)	*ns*	0 (0)	0 (0)	*ns*
normocellularity	3 (30)	1 (10)	*ns*	3 (100)	5 (83)	*ns*
normal BM blast counts <5%	10 (100)	3 (10)	***p =* 0.003**	3 (100)	6 (100)	*ns*
cellularity [%]	50 (30–90)	45 (20–90)	*ns*	n. a.	n. a.	
blasts [%]	3 (0–4)	9 (2–15)	***p* = 0.003**	n. a.	n. a.	
**Specifications of MDS patients**				**Specification of Controls**		
**Karyotype (IPSS-R)**				Suspected transfusion reaction	Hodgkin lymphoma	
very good/good risk	6 (60)	5 (50)	*ns*	localized follicular lymphoma	diffuse large B-cell lymphoma	
intermediate risk	4 (40)	2 (20)	*ns*	Solitary plasmocytoma	post-transplant lympho- proliferative disorder	
poor/very poor risk	0 (0)	3 (30)	*ns*		Pulmonary adenocarcinoma	
**MDS-CI**					AIDS with cerebral toxoplasmosis	
low risk	5 (50)	5 (50)	*ns*		multicentric Castleman disease	
intermediate risk	5 (50)	2 (20)	*ns*			
high risk	0 (0)	3 (30)	*ns*			
**Treatment received before** **assessment**						
red blood cell transfusion	4 (40)	7 (70)	*ns*			
platelet transfusion	0 (0)	1 (10)	*ns*			
lenalidomide	0 (0)	1 (10) ^1^	*ns*			

Medians with range are shown for age, cellularity, and blasts. Means with standard-deviations (SD) are shown for blood count and blood chemistry. Absolute numbers with percentages are shown for gender and bone marrow features (except cellularity and blasts), karyotype, MDS-CI, and treatment received before assessment. *p*-values were calculated using the Mann–Whitney U test for ordinal-scaled data and Fisher’s exact test for contingency tables (gender, bone marrow features except for cellularity, blasts). Significance was determined at *p* < 0.05 (bold). More details can be found in Table S1.

**Table 2:** Expression of immune-related biomarkers in MDS patients and controls.

Biomarkers	MDS Patients			Controls		
	Lower Risk (*n* = 10)	Higher Risk (*n* = 10)		Non-Inflammatory (*n* = 3)	Inflammatory (*n* = 6)	
**Univariate Analysis**						
**Casp1**	9 (1–20)	4.25 (1.5–17.5)	*p* = 0.054	0 (0–7.5)	15.5 (9–22)	***p* = 0.028**
**PD1**	3 (0–10)	2.5 (1–10)	*p* = 0.784	1 (1–10)	4 (1–10)	*p* = 0.680
**PD-L1**	1.5 (0–12)	2.75 (1–21)	*p* = 0.238	4 (2–5)	10.5 (2–21)	*p* = 0.300
**Multivariate Analysis**						
**Casp1**						***p* = 0.003**
	X	X				*p adj* = 0.48
	X			X		*p adj* = 0.23
	X				X	*p adj* = 0.75
		X		X		*p adj* = 1.00
		X			X	***p adj =* 0.01**
				X	X	***p adj =* 0.01**
**PD1**						*p* = 0.895
	X	X				*p adj =* 1.00
	X			X		*p adj =* 1.00
	X				X	*p adj =* 1.00
		X		X		*p adj =* 1.00
		X			X	*p adj =* 1.00
				X	X	*p adj =* 1.00
**PD-L1**						*p =* 0.133
	X	X				*p adj =* 1.00
	X			X		*p adj =* 1.00
	X				X	*p adj* = 0.11
		X		X		*p adj =* 1.00
		X			X	*p adj* = 1.00
				X	X	*p adj =* 1.00

H-scores are shown as median with ranges for the univariate analysis, which was performed with Mann–Whitney U test. Multivariate analysis was done by Kruskal–Wallis test followed by post-hoc Dunn test with Bonferroni correction (*p* adj for each comparison). Significance was determined at *p* and *p adj* < 0.05 (bold).

**Table 3:** Follow-up and endpoints in MDS patients.

	**Lower Risk (*n* = 10)**	**Higher Risk (*n* = 10)**	
**Follow-Up (Days), Median (Range)**	648.5 (107–1799)	471 (15–3576)	*ns*
**Treatment, *n* (%)**			
**Disease-Modifying Treatment (DMT)**	8 (80)	9 (90)	*ns*
*hypomethylating agents (HMA)*	4 (40)	7 (70)	*ns*
*allo-HSCT*	1 (10)	3 (30)	*ns*
*Other DMTs*	4 (40)	5 (50)	*ns*
red blood cell transfusions	8 (80)	9 (90)	*ns*
platelet transfusions	7 (70)	6 (60)	*ns*
**Fate, *n* (%)**			
alive	2 (20)	4 (40)	*ns*
*Allo-HSCT survivor*	1 (50)	3 (75)	*ns*
dead	8 (80)	6 (60)	*ns*
*MDS-related death*	6 (75)	6 (100)	*ns*
*not MDS-related death*	1 (13)	0 (0)	*ns*
*unknown death*	1 (13)	0 (0)	*ns*
**Endpoints (days), median (range)**			
time to treatment (TTT)	289 (54–1015)	55.5 (8–2632)	***p* = 0.04**
progression-free survival (PFS)	429 (88–1264)	161.5 (15–3576)	*ns*
leukemia-free survival (LFS)	951 (107–1799)	143.5 (15–616)	*ns*
overall survival (OS)	955 (107–1799)	170.5 (15–625)	*ns*

Other disease-modifying treatments (other DMTs) included cyclosporine/thymoglobulin (*n* = 3), lenalidomide (*n* = 2), hydroxyurea (*n* = 2), and cytarabine/idarubicin (*n* = 1). Patients undergoing allo-HSCT were censored at time of transplantation for PFS, LFS, and OS. *p*-values were calculated with Fisher’s exact test for contingency tables and the Kaplan–Meier estimator with log-rank test was used for survival rates. Significance was determined at *p* < 0.05 (bold). The survival curve of TTT can be found in Figure S3.

## Data Availability

The datasets used and/or analyzed during the current study are available from the corresponding author on reasonable request.

## Supplementary Materials

The following are available online at https://www.mdpi.com/article/10.3390/cancers13225712/s1, Figure S1: Immunohistochemical stainings from representative MDS patients, Figure S2: Immunohistochemical stainings from representative controls, Figure S3: TTT in lower and higher risk MDS patients, Figure S4: Predictive power of Casp1 as classifier for MDS disease stages, Table S1: Detailed clinical data from MDS patients and controls, Table S2: Correlations of immune-related biomarkers in MDS patients and controls, Table S3: Correlations between immune-related biomarkers and relevant laboratory values in MDS patients, Table S4: Correlations of immune-related biomarkers with survival endpoints in MDS patients.
