# Supplementary material for: Diagnostic and Prognostic Implications of Caspase-1 and PD-L1 Co-Expression Patterns in Myelodysplastic Syndromes
Source: Cancers (Basel). 2021 Nov 15;13(22):5712. doi: 10.3390/cancers13225712 (PMC8616142; doi:10.3390/cancers13225712)
Supplement: Supplementary file 1 [file cancers-13-05712-s001.zip › cancers-1413247-supplementary.pdf]

Supplemental Material

Suppl. Table S1: Detailed clinical data from MDS patients and controls.

MDS patients

	N	IPSS-R	Casp1	PD1	PD-L1	C/P	Hb	ANC	Mo	Tc	CRP	bBM	cBM	first DMT	allo HSCT	OS
Lower risk	1	4	8	4	12	0.67	75	1.96	1.66	54	60	2	▲	AZA	Yes	Alive
	2*	3.5	1	10	5.5	0.18	82	0.86	0.01	85	11	2	▼	None*	No	Alive
	3#	2.5	20	10	1	20	155	4.95	0.82	87	35	3	=▲	AZA	No	1697
	4	4	9	2	3.5	2.57	64	0.23	0.03	42	2	3	▼	CY	No	356
	5	3	11.5	5	2	5.75	113	3.51	0.64	110	3	4	▲	AZA	No	1343
	6	2	9	0	0	x	102	1.27	0.37	6	3	0	=	Other	No	151
	7	2	13.5	1	1	13.5	100	4.11	0.32	280	1.7	4	▲	Not known	No	1247
	8	3.5	18	1	0.5	36	76	3.13	0.25	434	11	4	=	LEN	No	107
	9*	4	2	2	1	2	137	1.84	0.28	45	3	3	=	AZA	No	663
	10	4	8.5	4	5	1.7	91	1.32	0.16	67	32	4	=▲	Not known	No	1799
Higher risk	11	7	1.5	1	10	0.15	88	0.17	0.22	13	148	13	▲	LEN	No	183
	12#	6	4.5	10	21	0.21	74	0.71	0.05	22	6	7	▲	CYT/IDA	Yes	Alive
	13	4.5	6.5	1	3	2.17	128	1.53	0.1	57	0	4	▲	AZA	No	Alive
	14	4.5	6	10	3.5	1.71	91	0.15	0.14	175	14	8	=▲	AZA	Yes	Alive
	15	4.5	3.5	1	1.5	2.33	80	1.3	0.25	7	3	2	=	CY/TG	Yes	Alive
	16*	9	17.5	4	2	8.75	68	1.93	0.3	64	4	15	=▲	AZA	No	138
	17	7	1.5	1	2.5	0.6	70	0.21	0	23	20	15	=▲	AZA	No	321
	18	5.5	7.5	1	2	3.75	111	2.32	0.73	96	18	11	n.a.	None	No	15
	19	5	3.5	4	1	3.5	90	4.17	0.08	78	47	8	▲	AZA	No	158
	20	6.5	4	4	7	0.57	69	1.1	0.14	229	3	10	=▲	AZA	No	625

Controls

	N	Casp1	PD1	PD-L1	C/P	Hb	ANC	Mo	Tc	CRP	bBM	cBM	Conditions
Non-inflammatory	C1	0	10	4	0	110	4.61	0.37	191	3	<5	=	Suspected transfusion reaction
	C2#	0	1	2	0	n.a.	n.a.	n.a.	n.a.	n.a.	<5	=	Localized (extramedullary) follicular lymphoma
	C3	7.5	1	5	1.50	153	n.a.	n.a.	310	4	<5	=	Solitary (extramedullary) plasmocytoma .
Inflammatory	C4	9	10	2	4.50	139	n.a.	n.a.	331	115	<5	=	Hodgkin lymphoma
	C5	14	6	6	2.33	124	22.01	1.92	334	111	<5	=	Pulmonary adenocarcinoma
	C6	22	1	15	1.47	116	4.08	n.a.	226	3	<5	n.a.	Diffuse large B-cell lymphoma
	C7	15	1	16.5	0.91	82	3.73	n.a.	29	25	<5	=▲	AIDS with cerebral toxoplasmosis
	C8	16	2	2.5	6.40	79	8.36	n.a.	502	97	<5	=▲	Post-transplant lymphoproliferative disorder
	C9#	19	10	21	0.91	84	4.55	n.a.	107	58	<5	▲	Multicentric Castleman Disease

Patient and control numbers [N]; International Prognostic Scoring System revised [IPSS-R]; Casp1: Caspase1 [H-score]; PD1: Programmed cell death protein 1 [H-score]; PD-L1: Programmed cell death 1 ligand 1 [H-score]; C/P: Casp1/PD-L1 Ratio; Hb: hemoglobin [g/L]; ANC: absolute neutrophile counts [10^9/L]; Mo: monocytes [10^9/L]; Tc: thrombocytes [10^9/L]; CRP: C-reactive protein [mg/L]; bBM: BM blasts [%], cBM: cellularity of BM (▼: hypocellular, =: normocellular, ▲: hypercellular). DMT: disease-modifying treatment (AZA: azacytidine; LEN: lenalidomide;

CYT/IDA: cytarabine/idarubicin; CY/TG: cyclosporine/ thymoglobulin; *: on hold for allo HSCT); allo HSCT: allogenic hematopoietic stem-cell transplantation, OS: overall survival [days].

IHC pictures of patients/controls with # are show in Suppl. Figure 1 and 2, respectively. Patients with * had unexpected patterns and were investigated in more detail. Non-available (n.a.) laboratory values in controls are from primary/secondary care facility BM specimen examined in our tertiary care hospital.

Suppl. Table S2: Correlations of immune-related biomarkers in MDS patients and controls.

	MDS patients			Controls		
	**Casp1**	**PD1**	**PD-L1**	**Casp1**	**PD1**	**PD-L1**
**Casp1**		rho = 0.01 *p = 0.98*	rho = -0.41 *p = 0.07*		rho = -0.06 *p = 0.88*	rho = 0.64 *p = 0.06*
**PD1**			rho = 0.41 *p = 0.07*			rho = -0.07 *p = 0.85*

Regression analysis was performed with Spearman's rank correlation and significance determined using Bonferroni correction (dark green: $p<0.01$; light green: $p<0.05$; orange: $p<0.10$; red: $p>0.10$).

Suppl. Table S3: Correlations between immune-related biomarkers and relevant laboratory values in MDS patients.

	Casp1+	PD1+	PD-L1+
Neutrophils [10^{-9}/l]	*rho = 0.57 p = 0.009*		*rho = - 0.58 p = 0.007*
Monocytes [10^{-9}/l]	*rho = 0.52 P = 0.02*		
Hemoglobin [g/l]			*rho= - 0.45 p = 0.046*
Thrombocytes [10^{-9}/l]			
BM cellularity [%]			
BM blasts [%]			
CRP [mg/L]			

Dark green: p<0.01; Light green: p<0.05; Orange: p<0.10; Red: p>0.10

Regression analysis was performed using Spearman's rank correlation and probabilities adjusted using Bonferroni correction.

Suppl. Table S4: Correlations of immune-related biomarkers with survival endpoints in MDS patients.

	**Casp1$_{high}$ (n=10)**	**Casp1$_{low}$ (n=10)**		**PD1$_{high}$ (n=10)**	**PD1$_{low}$ (n=10)**		**PD-L1$_{high}$ (n=10)**	**PD-L1$_{low}$ (n=10)**	
**Time to treatment (TTT)**	123 (28-1015)	81.5 (8-2632)	*ns*	98.5 (8-1015)	73 (9-2632)	*ns*	81.5 (9-123)	289 (8-2632)	*ns*
**Progression-free survival (PFS)**	253.5 (15-1264)	271 (73-3576)	*ns*	559 (88-1264)	205.5 (15-3576)	*ns*	308 (73-1264)	216.5 (15-3576)	*ns*
**Leukemia-free survival (LFS)**	356 (15-1799)	260 (130-655)	*ns*	979.5 (138-1799)	205.5 (15-1247)	*ns*	356 (130-1799)	151 (15-1519)	*ns*
**Overall survival (OS)**	356 (15-1799)	321 (158-663)	*ns*	984 (138-1799)	252 (15-1247)	*ns*	356 (183-1799)	158 (15-1697)	*ns*

Median with ranges in days are shown for all parameters. Patients were stratified according to the median H-score of Casp1 (7), PD1 (3), and PD-L1 (2.25) in high and low expressor groups. Kaplan-Meier estimator with log-rank test was used for the survival analysis with censoring for allo HSCT at time of transplantation. Significance was determined at $p<0.05$.

Suppl. Figure S1: Immunohistochemical stainings from representative MDS patients.

A: Lower risk MDS patient (3) with Casp1$_{high}$/PD-L1$_{low}$ pattern

(H-scores Casp1/PD-L1: 20/1)

Casp1

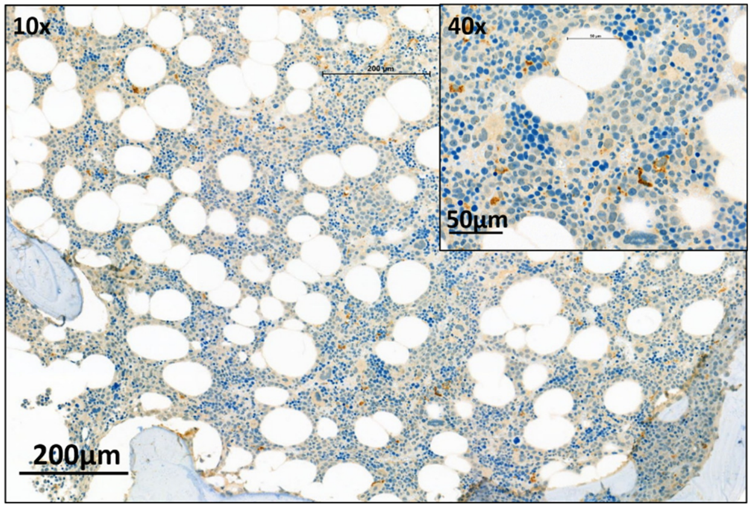

PD-L1

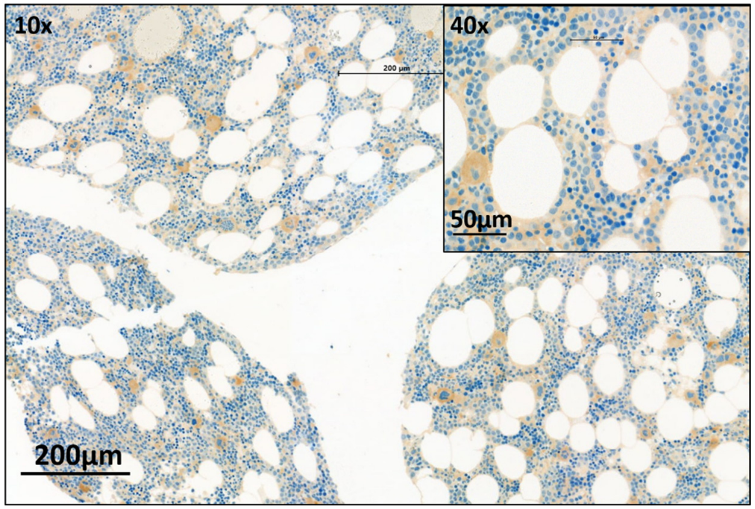

B: Higher risk MDS patient (12) with Casp1$_{low}$/PD-L1$_{high}$ pattern

(H-scores Casp1/PD-L1: 4.5/21)

Casp1

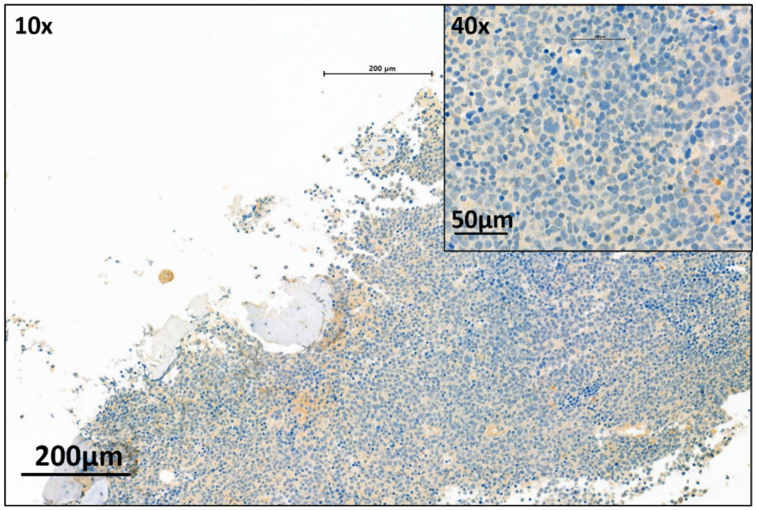

PD-L1

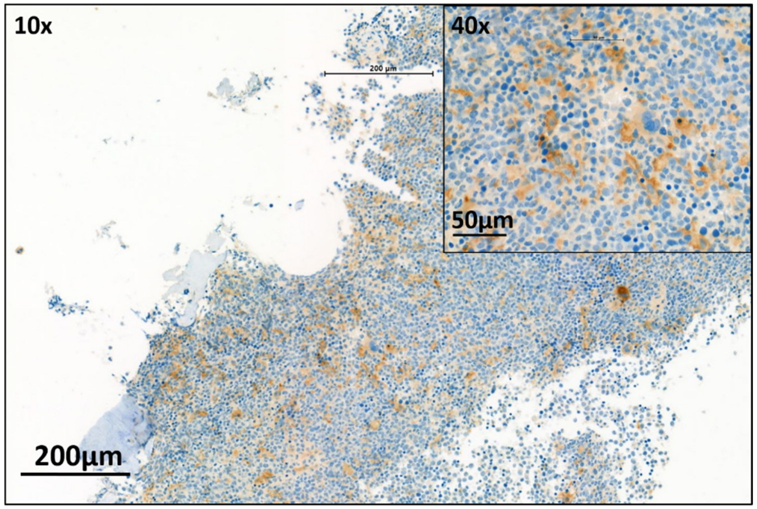

Patient numbers according to Suppl. Table 1 (see patients with #).

Suppl. Figure S2: Immunohistochemical stainings from representative controls.

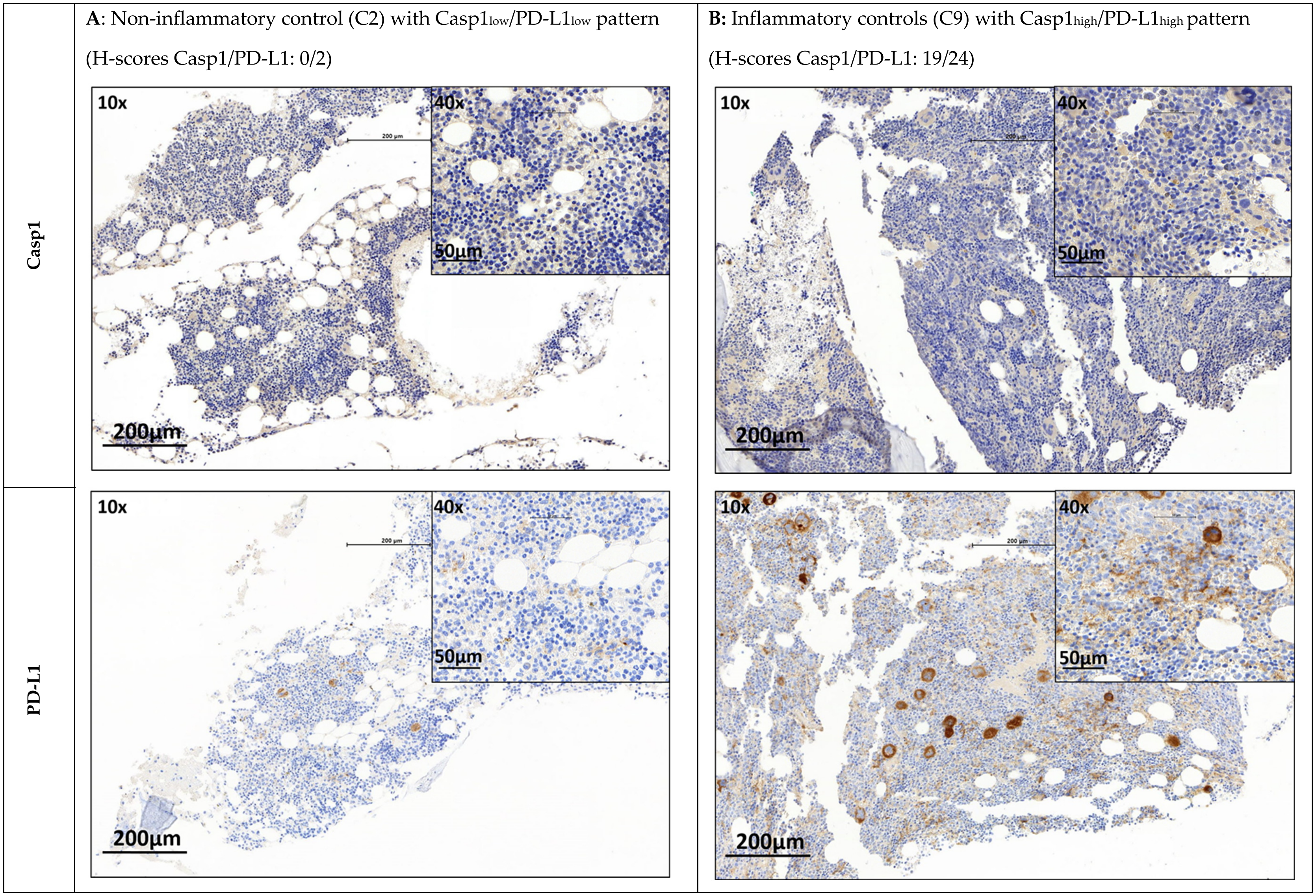

Numbers of controls according to Suppl. Table 1 (see controls with #).

Suppl. Figure S3: TTT in lower and higher risk MDS patients.

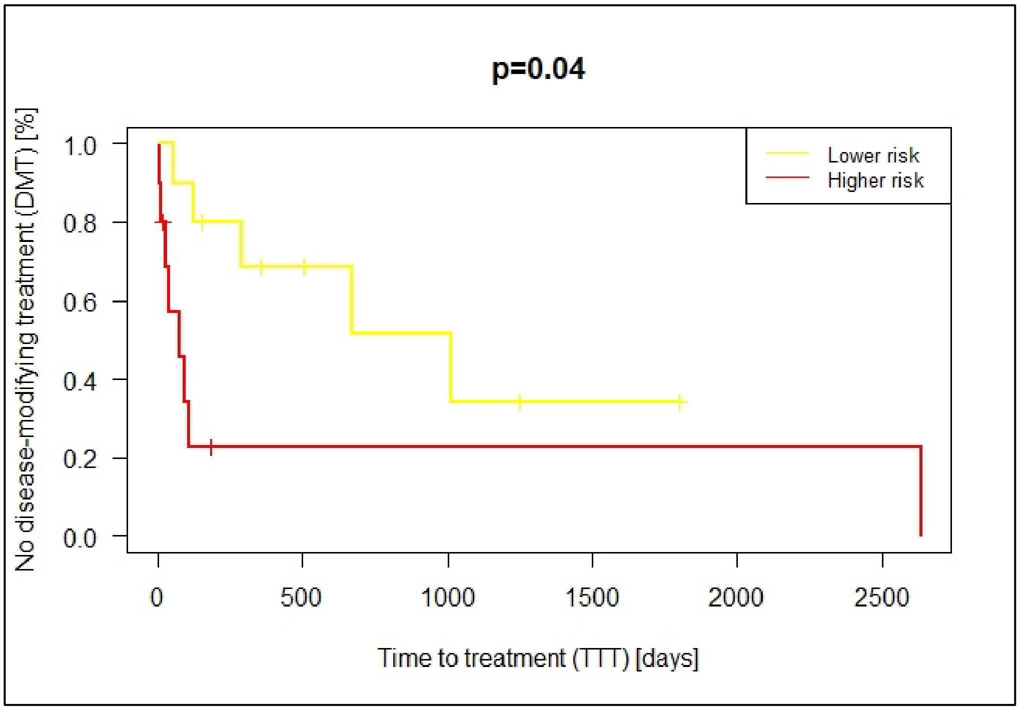

First disease-modifying treatment (DMT) as shown in Suppl. table 1. DMTs included HMA (n=11), allo HSCT (n=4), Cyclosporine/Thymoglobulin (n=3), Lenalidomide (n=2), Hydroxyurea (n=2) and Cytarabine/Idarubicine (n=1). Log rank test was used with significance determined at $p<0.05$.

Suppl. Figure S4: Predictive power of Casp1 as classifier for MDS disease stages.

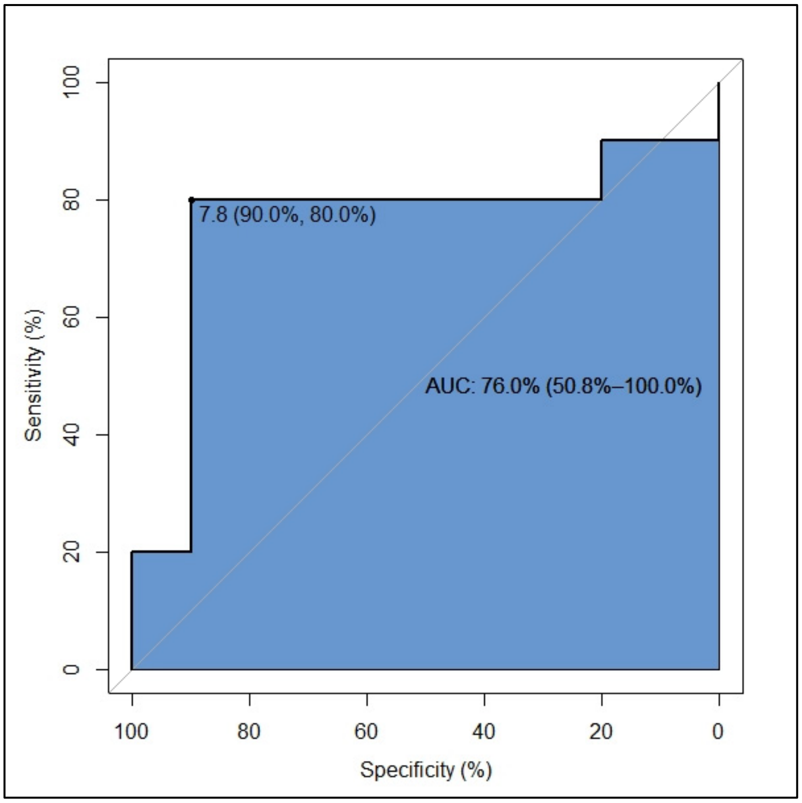

Receiver operating characteristic (ROC) analysis of Casp1 expression as classifier for lower and higher risk MDS disease stage. Casp1 H-score of 7.8 was used as cut-off, which resulted in a specificity of 90% and sensitivity of 80%. Due to low patient numbers, the area under the curve (AUC) resulted in a large 95% confidence interval.
